# Membrane domain formation—a key factor for targeted intracellular drug delivery

**DOI:** 10.3389/fphys.2014.00462

**Published:** 2014-12-02

**Authors:** Dušan Popov-Čeleketić, Paul M. P. van Bergen en Henegouwen

**Affiliations:** Division of Cell Biology, Department of Biology, Faculty of Science, Utrecht UniversityUtrecht, Netherlands

**Keywords:** membrane domain, receptor mediated endocytosis, EGFR, receptor clustering, drug delivery, cancer therapy

## Abstract

Protein molecules, toxins and viruses internalize into the cell via receptor-mediated endocytosis (RME) using specific proteins and lipids in the plasma membrane. The plasma membrane is a barrier for many pharmaceutical agents to enter into the cytoplasm of target cells. In the case of cancer cells, tissue-specific biomarkers in the plasma membrane, like cancer-specific growth factor receptors, could be excellent candidates for RME-dependent drug delivery. Recent data suggest that agent binding to these receptors at the cell surface, resulting in membrane domain formation by receptor clustering, can be used for the initiation of RME. As a result, these pharmaceutical agents are internalized into the cells and follow different routes until they reach their final intracellular targets like lysosomes or Golgi. We propose that clustering induced formation of plasma membrane microdomains enriched in receptors, sphingolipids, and inositol lipids, leads to membrane bending which functions as the onset of RME. In this review we will focus on the role of domain formation in RME and discuss potential applications for targeted intracellular drug delivery.

## Introduction

Receptor-mediated endocytosis (RME) is a way of internalization of larger molecules, toxins and viruses into the cell using specific proteins and lipids in the plasma membrane. Whereas a majority of small molecules can diffuse through the plasma membrane (PM), larger cargo needs energy-dependent assistance of both proteins and lipids in the PM to get into the cell. Upon binding of the ligand to the receptor on the cell surface, a signaling cascade is activated, leading to the invagination of the membrane, followed by the formation of the vesicle. The vesicle then pinches of the membrane and, following one of the endocytic pathways, gets transferred to a specific intracellular location.

Due to high specificity and efficiency, RME presents itself as an excellent system for drug delivery of various therapeutical compounds/agents. In cancer therapy, receptors used as targets for different pharmaceutical agents comprise mostly receptor tyrosine kinases and the transferrin receptor, as these proteins are overexpressed in a number of tumors (Zaki and Tirelli, 2010). Other receptors that are investigated for targeted drug delivery, albeit in a less extent, comprise vitamin receptors, like folate and riboflavin receptors, lectin receptors, receptors for cell adhesion molecules, etc.

Mechanistic details of receptor-mediated drug delivery are studied mostly with a focus on the interaction between a receptor and a corresponding ligand, as well as on the specific endocytic pathway. Recently, more attention is being paid to the changes in the lipid environment. The elucidation of this aspect of RME is not only an important fundamental question but could also improve drug delivery. The changes in the lipid environment during RME refer not only to larger segments of PM that get internalized with ligand bound receptors, but also to the formation of membrane domains of distinct features that are present in the closest vicinity of the receptors.

## Domains in the plasma membrane

Plasma membrane (PM) is a dynamic mixture of proteins and lipids that delineates the cell and enables the communication with the environment. Proteins that function within PM are integrated in or associated with the lipid bilayer composed of three major classes of lipids: glycerophospholipids, sphingolipids, and sterols (van Meer, 2005). Since Singer-Nicolson's model of the plasma membrane (Singer and Nicolson, 1972) our understanding of the organization of PM and the regulation of its function has drastically evolved (Engelman, 2005; Simons and Gerl, 2010; Kusumi et al., 2012). The plasma membrane is no longer depicted as a fluid lipid carpet with mosaically distributed proteins associated with or spanning the lipid bilayer. Instead, the plasma membrane can be seen more as a city map where protein-lipid ensembles of specific localization, size and orientation are non-homogenously distributed in distinct regions, often termed as membrane domains. The membrane domain should therefore be understood as a general term comprising various suborganellar constructions of distinct features found in the membrane.

These non-homogenously distributed ensembles of the plasma membrane are found in membranes of a number of organisms including bacteria (Maddock and Shapiro, 1993), yeast (Malínská et al., 2003), and in a range of mammalian cells, including cancer cells (Staubach and Hanisch, 2011; George and Wu, 2012). Ergosterol rich microcompartment of Can1 (MCC) in *S. cerevisiae* was shown to be composed of specific lipids, being enriched in ergosterol in particular, and relatively a well-defined protein content of at least 21 proteins (Grossmann et al., 2007, 2008). The MCC proteins are rarely found in the other membrane microdomain, the microcompartment of Pma1 (MCP). Both MCC and MCP are relatively large (over 300 nm), and can be studied using various methods of optical microscopy. Analysis of MCC using electron microscopy revealed that MCC are structurally organized into furrow-like invaginations (Stradalova et al., 2009). This structure is maintained by BAR domain proteins Pil1 and Lsp1 (Ziółkowska et al., 2011). These banana-shaped proteins associate with the PM on the cytosolic side inducing a geometrical curvature of the membrane.

Lipid rafts in mammalian cells are still a matter of controversy. They were initially identified as cholesterol and sphingolipids-enriched microdomains found in detergent insoluble membrane fractions. It was shown that these fractions contain increased levels of glycophosphatidylinositol-anchored proteins and a number of signaling molecules suggesting that rafts play significant roles in immunity, signaling, organization of cytoskeleton, etc. (Brown and Rose, 1992; Simons and Ikonen, 1997; Simons and Toomre, 2000; Harris and Siu, 2002). The existence of lipid rafts was, however, difficult to be proven *in vivo*. Detergent extraction that was used to prove the existence of lipid rafts could not be standardized and the results obtained by different groups showed large variations (Munro, 2003). With the development of novel techniques, like super resolution light microscopy and fluorescence resonance energy transfer (FRET), nanoclusters containing raft proteins were shown to exist *in vivo*, yet the detailed mechanism of their formation and the exact role in different cellular processes is still being investigated (Lingwood and Simons, 2009; Simons and Gerl, 2010).

Lipid raft proteins and lipids are present in the vesicles of distinct endocytic pathways. Clathrin independent endocytosis (CIE) occurs mostly via formation of lipid rafts (Hansen and Nichols, 2009; El-Sayed and Harashima, 2013). For example, caveolae-mediated endocytosis, a type of CIE, requires direct interaction between protein caveolin and cholesterol in the lipid rafts for the formation of the vesicles (Murata et al., 1995; Smart et al., 1999). Conversely, clathrin-mediated endocytosis (CME) does not appear to be initiated within the lipid rafts, as the former do not enter clathrin-coated pits (Nichols, 2003).

In addition to lipid rafts, another type of membrane domain was suggested to exist in the PM. Already during 1970s, it was observed that the proteins and lipids diffuse much slower (up to 20 times) *in vivo* than in artificial membranes (Axelrod et al., 1976; Baier et al., 2009). In erythrocyte cells lacking the spectrin-based cortical network an increase in diffusion coefficient of a membrane protein was reported (Sheetz et al., 1980). Studying the trajectories of individual molecules using single particle tracking demonstrated that several proteins from the PM diffuse within a confined moving surface (Kusumi et al., 1993). In kidney cells with disrupted cytoskeleton, the confinement space and the diffusion rates of membrane proteins were shown to be increased (Sako and Kusumi, 1995). Diffusion rates of phospholipids increased up to nine-fold when actin filaments were obstructed (Fujiwara et al., 2002). It is hypothesized that actin forms a membrane skeleton on the cytosolic side of PM that interacts with integrated membrane proteins and forms membrane compartments of the size of 40–300 nm (Kusumi et al., 2012).

Different membrane microdomains containing various combinations of membrane proteins and lipids have been identified throughout the years. These domains do not necessarily fit the definition of either rafts or actin restricted membrane compartments. They are more or less transient and of various sizes, and are characterized based on the interactions between proteins and lipids. In addition, novel types of microdomains are generated as a response to exogenous and endogenous signals. An example could be the formation of microdomains consisting of peptide-lipid complexes of high molecular weight as a reaction to increased levels of human islet amyloid polypeptide during the development of diabetes type II (Guo et al., 2014).

To get a full understanding of such a diversity of membrane microdomains and a complex function of the PM one must take into account biophysical features of both proteins and lipids. On the one hand, specific protein localization and confinement depend on lipid composition and distribution. On the other hand, lipid moiety influences the function of proteins through several biophysical parameters like the charge of lipid polar heads (Kalli et al., 2013) and membrane voltage (Bezanilla, 2008), as well as steric effects. Latter effects are exerted by individual lipid molecules, particularly cholesterol and ergosterol, in mammalian and yeast cells, respectively (Sheets et al., 1999; Kwik et al., 2003). In a more general manner, steric effects are exerted through the geometrical changes of membrane curvature. Regions in the plasma membrane with different curvatures have different protein and lipid composition. Changes in geometrical membrane curvature were suggested to play a role in regulating the function and distribution of both proteins and lipids (Sorre et al., 2009; Henne et al., 2010; Antonny, 2011; Domanov et al., 2011; Tonnesen et al., 2014). The occurrence of a very fast bending of the membrane during RME could therefore play a role in dynamic regulation of transmembrane protein function. This speculation still needs to be investigated.

## EGFR as a model for the mechanism of the receptor clustering

The organization of the PM in a number of essential cellular processes is studied using a number of different well-defined proteins and lipids. Receptor tyrosine kinases (RTK) are often used as model proteins for studying receptor clustering and the formation of membrane domains. The epidermal growth factor receptor (EGFR) belongs to ErbB family of RTK and is a crucial member of signaling networks regulating cell proliferation. Other members of ErbB family are HER2-4. The mechanisms and the biology of ErbB proteins are complex; there are 11 different growth factors serving as ligands for 4 ErbB receptors (Linggi and Carpenter, 2006). ErbB proteins are crucial in the embryonic stage for the development of nervous system, and necessary for normal heart function (Lemmon et al., 2014). All members of ErbB family are involved with the development of different types of cancer and were successfully used as targets in cancer therapy (Arteaga and Engelman, 2014).

**Figure 1:**
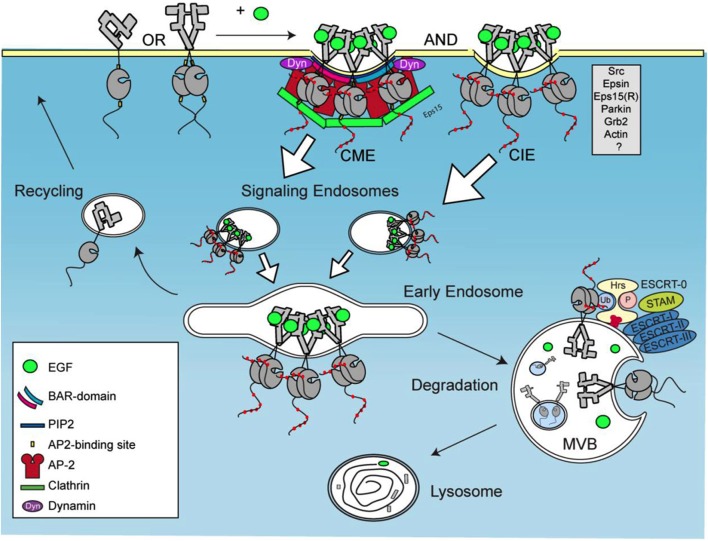
**Trafficking of activated EGFR**. EGFR clustering occurs upon activation by EGF, followed by internalization via both clathrin-mediated and clathrin independent endocytosis, CME and CIE, respectively. This leads to formation of signaling endosomes that fuse into early endosomes and multivesicular bodies (MVB). Second type of EGFR internalization occurs in MVBs via ESCRT complex.

EGFR is a single transmembrane domain with N-terminus exposed to the extracellular matrix and a C-terminus with a kinase domain exposed to cytoplasm (Jorissen et al., 2003). The N-terminal part of EGFR consists of four domains involved in ligand binding and receptor dimerization. Ligand binding to EGFR leads to receptor dimerization and transphosphorylation, as well as to the formation of receptor clusters (Clayton, 2005; Saffarian et al., 2007). Receptor clustering within a specific membrane domain is crucial in several signaling pathways (Minguet et al., 2007; Nikolaev et al., 2010). Receptor clustering that occurs upon binding of EGF to EGFR depends on its kinase activity (Hofman et al., 2010). Moreover, EGF was shown to induce the formation of EGFR nanoclusters depending on the activity of phospholipase D2 (Ariotti et al., 2010). Upon its activation, EGFR is quickly internalized (see Figure 1). After internalization and endosomal sorting EGFR is either degraded in lysosomes or recycled back to PM (Sorkin and Goh, 2009). Under physiological concentrations of ligand, the internalization of EGFR occurs mainly via CME. In the presence of increased levels of EGF and the overexpression of EGFR, the ubiquitination of the receptor is increased and the internalization occurs via CIE to a larger extent (Carpentier et al., 1982; Wiley, 1988; Sigismund et al., 2005). CIE appears to be a default pathway for the degradation of EGFR whereas CME mostly leads to recycling of EGFR into the PM (Sigismund et al., 2008).

According to crystal structures the extracellular part of EGFR is responsible for EGFR dimerization (Ferguson, 2008). Dimerization of EGFR is also regulated by its transmembrane domain, as this domain contains two GxxxG dimerization motifs, one closer to N terminus, and one closer to the C terminus. Molecular dynamics (MD) simulations suggest that in ligand-bound dimers, conformational changes of the extracellular domains favor dimerization of the transmembrane helices near their N termini (Arkhipov et al., 2013). Site specific cystein crosslinking studies showed that crosslinks could be made within the N-terminal GxxxG motif (Lu et al., 2010). The N-terminal GxxxG dimerization motif was found to be essential for clustering induced internalization (Heukers et al., 2013).

In reconstitution studies it was shown that cholesterol predominantly stabilizes the dimerization of the N-terminal GxxxG motif (Jones et al., 1997). We favor the model where the formation of EGFR clusters results in the assembly of ganglioside GM1 and cholesterol enriched membrane domains. This stabilizes the formation of N-terminal transmembrane domain dimers, resulting in the bending of the membrane. As a result, local lipid pressure is reduced inviting the insertion of proteins with a BAR domain. The F-BAR domain proteins, such as FCH01/2, are among the first proteins that are recruited to the curved membrane, the site in the membrane where receptor internalization is initiated (Henne et al., 2010; McMahon and Boucrot, 2011; Taylor et al., 2011). Models of mechanisms of membrane domain formation and subsequent membrane curvature formation as the effect of specific protein clustering were suggested for several proteins. The most prominent example is the binding and internalization of Shiga toxin from *Shigella dysenteriae*. Association of Shiga toxin, with its glycolipid receptor leads to the formation of clusters that induce negative curvature on the membrane, resulting in the formation of narrow invaginations (Römer et al., 2007). Shiga toxin consists of two subunits, where the bigger one has homopentameric structure. Similar models where pentameric protein scaffolds together with glycosphingolipids like ganglioside GM1 induce plasma membrane curvature, followed by the endocytic uptake into cells, were also proposed for some viruses, like SV40 and norovirus (Ewers et al., 2009; Rydell et al., 2013). In addition, similarities between EGFR clustering model and the model of formation of MCC in yeast (Douglas et al., 2011) are more than evident.

## EGFR clustering and cancer therapy

Understanding the detailed mechanism of EGFR internalization is crucial regarding the connection of EGFR and cancer. Mutant forms and pathologically increased expression levels of EGFR and other members of ErbB family, HER2, ErbB3, and ErbB4 are implicated in cancer initiation and development. Thirty years ago mutant forms of EGFR and HER2 were linked to cancer cells in chicken and rat, respectively (Downward et al., 1984; Schechter et al., 1984). The evidence for the connection between ErbB family members and different forms of cancer, accumulated over the years, led to the development of different agents for the inhibition of EGFR as anti-cancer drugs (Cancer Genome Atlas Network, 2012; Lemmon et al., 2014). Inhibitors of ErbB family receptors have proven to be rather successful agents in cancer therapy. They include monoclonal antibodies (mAbs) like cetuximab (anti-EGFR) or trastuzumab (anti-HER2), small molecules that are inhibitors of kinase activity (lapatinib), or antibody-drug conjugates (Oliveira et al., 2006; Arteaga and Engelman, 2014).

Cetuximab binds to EGFR with higher affinity than EGF and induces internalization and degradation of the receptor. However, as cancer cells quickly adapt and become resistant to Cetuximab therapy, combination therapies that would be tailored to individual patients are proposed as a novel strategy for the treatment of EGFR-driven cancers (Arteaga and Engelman, 2014). One example is the targeting of multiple epitopes on EGFR simultaneously. Sym004, the mixture of two monoclonal anti-EGFR antibodies, induced removal of the receptor from the cancer cell surface by triggering EGFR internalization and degradation, and was effective in treatment of Cetuximab-resistant tumors (Pedersen et al., 2010; Iida et al., 2013). Triepitopic fusion protein was also shown to overcome acquired resistance to Cetuximab (Spangler et al., 2012). In addition, antibodies against EGFR conjugated to nanoparticles or liposomes carrying cytotoxic agents have been used for targeted drug delivery. Liposomes containing doxorubicin and conjugated on the surface with anti-EGFR antibody and folic acid targeted both EGFR and folate receptor with enhanced selectivity and reduced toxicity to healthy cells when compared to liposomes targeted to a single receptor (Saul et al., 2006).

In constant pursuit of more efficient agents for cancer detection and treatment novel approaches are being investigated. One example are nanobodies, variable domains of the heavy chain-only antibodies found in camels, lamas, alpacas and certain species of sharks (Hamers-Casterman et al., 1993). Nanobodies are 10 times smaller than antibodies, yet having strong and specific binding comparable to those of conventional antibodies. Nanobodies against EGFR developed in our group have shown several advantages in cancer detection when compared to mAbs (Oliveira et al., 2012, 2013). The accumulation in tumor was faster and distribution was more homogeneous, with a more rapid clearance of unbound molecules. Similar to mAb therapy, nanobody-drug conjugates are being developed for efficient drug delivery. First experiments with biparatopic nanobody that binds two different epitopes on EGFR, showed the induction of EGFR clusters in the plasma membrane, leading to the kinase independent internalization. This clustering induced internalization was completely dependent on the presence of the N-terminal dimerization motif. This is in agreement with the abovementioned model of interdependence between EGFR clustering and membrane microdomain formation. When these internalizing nanobodies were conjugated to a photosensitizer a higher toxicity was observed when compared to a conjugate consisting of a non-internalizing nanobody (Heukers et al., 2014). These results clearly showed the advantage of using internalizing nanobodies for the intracellular delivery of toxic compounds into cancer cells.

## Conclusive remarks

The majority of studies on protein lipid interactions were performed in artificial membranes *in vitro* and with MD simulations *in silico*. Tackling the questions connected to the topic using experiments *in vivo* have proven to be quite challenging and not rarely a subject of controversy. This refers in particular on the definition of membrane rafts. Novel single molecule methods enable studying various processes associated with PM *in vivo*. Alongside the development of technology and computer algorithms that allow observing dynamic process on the PM of living cells in high resolution, adequate model proteins and model lipids are being tested. Studying activation, clustering, internalization and trafficking of EGFR is a model system that allows us to tackle fundamental questions regarding lateral organization of lipids and proteins into membrane domains, as well as their structural and functional features.

Development of agents that would affect receptor clustering, either on the level of the receptor, or on the level of the organization of lipids in the membrane domain, was suggested as a novel approach in receptor targeting for therapeutic purposes (Bethani et al., 2010). Understanding the detailed mechanism of protein lipid interdependence during EGFR clustering is an example of a beautiful overlap of the fundamental and the pharmaceutical research. With the elucidation of this mechanism, more principles in the organization of the plasma membrane will be understood. These principles could further on be tested on other cell types as well as other organisms. At the same time, establishing exact mechanism and common principles of receptor clustering as onset of RME would be crucial in further development of ways of targeting and specific delivery of pharmaceutical agents.

### Conflict of interest statement

The authors declare that the research was conducted in the absence of any commercial or financial relationships that could be construed as a potential conflict of interest.
